# A Characterization of the *Manduca sexta* Serotonin Receptors in the Context of Olfactory Neuromodulation

**DOI:** 10.1371/journal.pone.0069422

**Published:** 2013-07-29

**Authors:** Andrew M. Dacks, Vincenzina Reale, Yeli Pi, Wujie Zhang, Joel B. Dacks, Alan J. Nighorn, Peter D. Evans

**Affiliations:** 1 Department of Biology, West Virginia University, Morgantown, West Virginia, United States of America; 2 Department of Neuroscience, the University of Arizona, Tucson, Arizona, United States of America; 3 Inositide Laboratory, the Babraham Institute, Cambridge, United Kingdom; 4 Department of Cell Biology, University of Alberta, Edmonton, Alberta, Canada; INSERM , UMR-S747, France

## Abstract

Neuromodulation, the alteration of individual neuron response properties, has dramatic consequences for neural network function and is a phenomenon observed across all brain regions and taxa. However, the mechanisms underlying neuromodulation are made complex by the diversity of neuromodulatory receptors expressed within a neural network. In this study we begin to examine the receptor basis for serotonergic neuromodulation in the antennal lobe of *Manduca sexta*. To this end we cloned all four known insect serotonin receptor types from *Manduca* (the Ms5HTRs). We used phylogenetic analyses to classify the Ms5HTRs and to establish their relationships to other insect serotonin receptors, other insect amine receptors and the vertebrate serotonin receptors. Pharmacological assays demonstrated that each Ms5HTR was selective for serotonin over other endogenous amines and that serotonin had a similar potency at all four Ms5HTRs. The pharmacological assays also identified several agonists and antagonists of the different Ms5HTRs. Finally, we found that the Ms5HT1A receptor was expressed in a subpopulation of GABAergic local interneurons suggesting that the Ms5HTRs are likely expressed heterogeneously within the antennal lobe based on functional neuronal subtype.

## Introduction

Our needs are in constant flux. The time of day, our level of stress and level of hunger are all examples of physiological contexts that represent dynamic internal and external environments, which in turn affect our perceptions and responses to the stimuli that we encounter. This context dependent adjustment of behavior is often accomplished via the release of neuromodulators in restricted areas of our nervous system by a small number of neurons. Neuromodulators adjust response properties of individual neurons and synaptic efficiency within neural circuits to alter the sensitivity, resolution and efficiency with which specific brain areas process information [1]. However, our understanding of the consequences of neuromodulation is limited by the complexity of the organization of neuromodulatory systems. A major contributor to this complexity is the large number of receptor subtypes for a given neuromodulator. For example, there are over a dozen serotonin receptor subtypes expressed in the vertebrate nervous system [2]. Furthermore, different populations of neurons within a given network sub-serve specific functions and may express different sets of receptors. Thus, neuromodulators have diverse effects on neural processing by differentially affecting distinct functional populations of neurons.

To gain insight into the organizational principles that underlie neuromodulation we began to study the receptor basis of serotonergic neuromodulation within the context of the primary olfactory neuropil (the antennal lobe or AL) of the moth *Manduca sexta*, a neural circuit with a parallel organization to the vertebrate olfactory bulb [3,4]. The effects of serotonin (5-HT) on olfactory processing at the level of individual neurons and neuronal ensembles have been studied extensively in *Manduca* [5–10] and have been found to be similar compared to other insects [11–13] and vertebrates [14–16]. The levels of 5-HT in the AL cycle throughout the day [6], reminiscent of the changes in the activity of serotonergic neurons in the Raphe nuclei with waking state [17–21]. In both vertebrates and insects, 5-HT enhances the responses of output neurons [5,6,8,12,14,15] and local interneurons [8,12,16]. Furthermore, 5-HT also enhances pre-synaptic inhibition of olfactory receptor neurons resulting in a gating of some odor-evoked responses in both vertebrates and insects [12,16]. Two of the four in sect 5-HT receptor subtypes have been cloned in *Manduca* [22], making the AL of *Manduca* well-suited for studying the organizational principles underlying serotonergic modulation of olfaction. In this study, we report the cloning of the two remaining 5-HT receptors from *Manduca* and examine the phylogenetic relationships and pharmacological characteristics of all four *Manduca* 5-HT receptors (the Ms5HTRs). We furthermore examined the expression patterns of the Ms5HT1A receptor within the AL of *Manduca*.

## Methods

### Receptor Cloning

**Figure 1:**
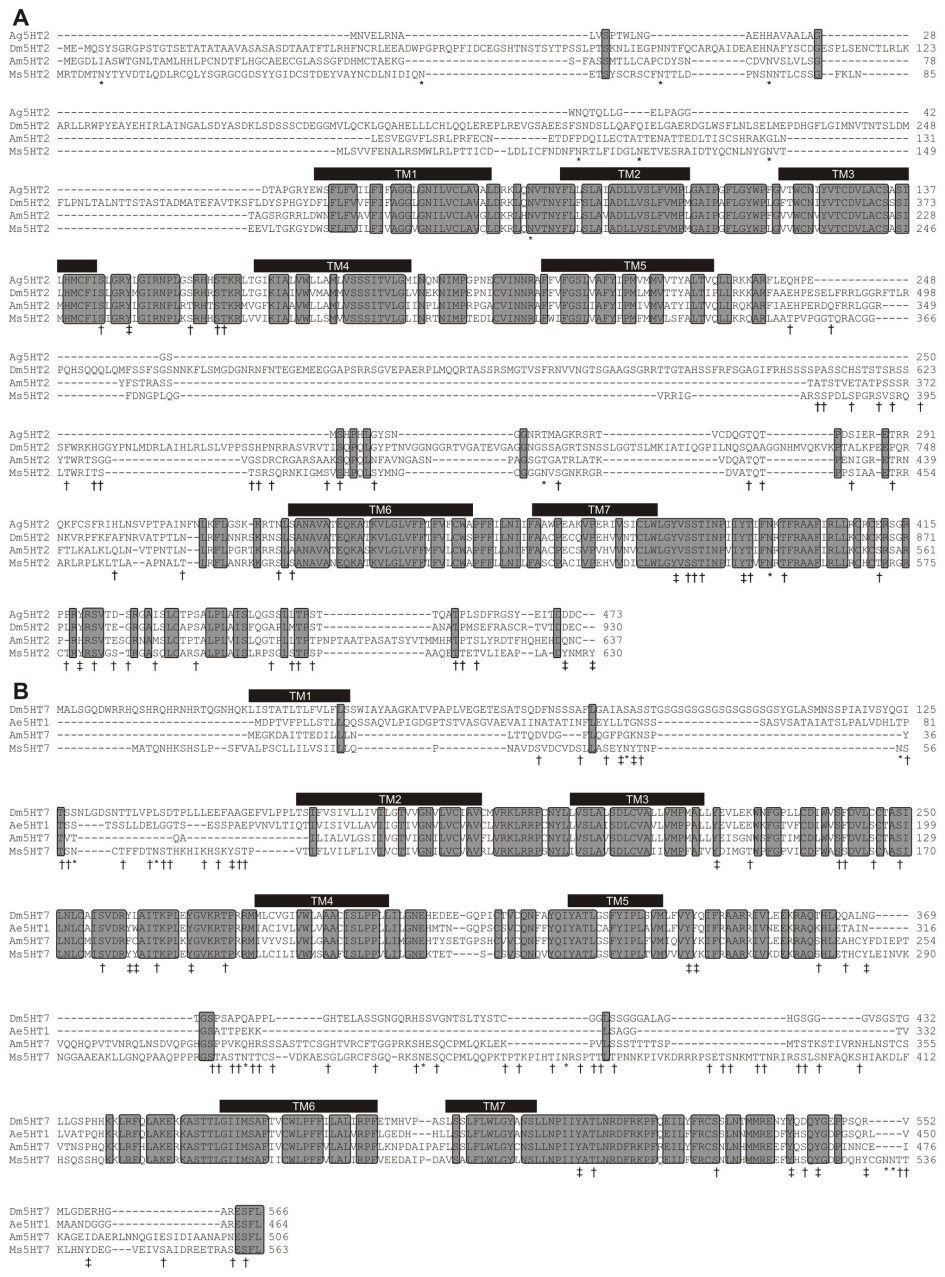
ClustalW sequence alignments for the Manduca 5-HT2 and 5-HT7 receptor homologues. Grey rectangles enclosing amino acid sequences indicate conserved sequence. For both receptors the putative 7 transmembrane domains are indicated by the black bars. Potential PKA/PKC phosphorylation sites are indicated by a “†”, tyrosine kinase phosphorylation sites by a “‡” and N-glycosylation sites by a “*”. (A) Alignment of the Ms5NT2R with 5-HT2 type receptors from Apis mellifera (Am5HT2), Drosophila melanogaster (DM5HT2) and Anopheles gambiae (Ag5HT2). (B) Alignment of the Ms5HT7 receptor with 5-HT7 receptors Drosophila melanogaster (Dm5HT7), Aedes aegypti (Ae5HT1) and A. mellifera (Am5HT7).

Degenerate PCR and RT-PCR were performed as described [22,23]. AL cDNA was isolated by cutting out the ALs only for mRNA extraction. Degenerate PCR primers were designed based on sequences from *Drosophila melanogaster*, *Aedes aegypti* and *Apis mellifera* using CODEHOP [24]. The degenerate PCR primer sequences used to clone the Ms5HT2 receptor were 5’ GAAGCTGCAGAACGCCACNAAYTAYTT 3’ and 5’ GAACACCATCATGAACATGGGNANRTARAA 3’, and 5' CGAGATCATGGGCAACTGGHBNTTYGG 3' and 5' GGTTGAACAGGGAGTTGCAGTANCCNARCCA 3' for the Ms5HT7 receptor. Brain and antennal lobe cDNA were generated using the Omniscript RT kit (Qiagen, Valencia, CA) and AccuPrime Pfx Supermix (Invitrogen) was used to generate initial fragments for the Ms5HT2 and 7 receptors. Rapid Amplification of cDNA Ends (RACE) was used to generate the full length sequence for the Ms5HT2 and Ms5HT7 receptors using the SMARTer PCR Synthesis Kit (Clontech, Mountain View, CA) to generate the cDNA with a universal tag sequence and Advantage 2 Polymerase Mix (Clontech) to generate 5’ and 3’ fragments using touchdown PCR. Sequence alignments in Figure 1A and B were constructed using the program ClustalW [25](http://npsa-pbil.ibcp.fr/cgi-bin/npsa_automat.pl?page=/NPSA/npsa_clustalw.html). The Genbank Accession numbers for the sequences used for the sequence alignments in Figure 1A and B were Ms5HT2 (JX891652), Ms5HT7 (JX878498), Ag5HT2 (XP_307953.2), Am5HT2 (CBX90120), Dm5HT7 (NP_524599.1), Ae5HT1 (XP_001651711.1), Am5HT7 (NP_001071289.1). Transmembrane domains were calculated as described previously [26] (ww.sbc.su.se/~miklos/DAS/)

### Phylogenetic Analyses

Homology searches were conducted via BLAST, using *Homo sapiens* sequences as queries for the vertebrate 5-HT receptors into the NCBI Genome database, and *Drosophila melanogaster* 5-HT receptor sequences for the in sect 5-HT receptor sequences into the NCBI non-redundant (nr) database. Species selected for the comparison of insect receptors were restricted to those species for which all four 5-HT receptors and several other amine receptors have been cloned. Vertebrate species selected for comparison were restricted to those in which a large number of the known 5-HT receptors have been annotated and span a wide phylogenetic breadth including representative fish (Danio rerio, Oreochromis niloticus), amphibians (Xenopus silurana), reptiles (Anolis carolinensis), birds (Gallus gallus, Meleagris gallopavo) and mammals (Ailuropoda melanoleuca, Homo sapiens, Rattus norvegicus, Ornithorhynchus anatinus). The search identified 5-HT, octopamine, tyramine and dopamine receptor homologs amongst the invertebrates and 5-HT receptor homologs amongst the vertebrate species. All candidates were subject to a reverse BLAST and from these, only candidates with e-values less than 2e-90 on the reverse BLAST were considered for further analysis to ensure the validity and reliability of the proposed homologous relationship. All of the accession numbers and abbreviations for the sequences used are listed in Table S1.

Three datasets were constructed comprised of insect and/or vertebrate amine receptors. Datasets were aligned using MUSCLE, then manually masked and trimmed using MacClade 4.08a, using only unambiguously homologous regions. The resulting alignments were then analyzed using ProtTest 1.3 v.10.2 to estimate the optimal model of sequence evolution for each individual dataset. The dataset containing biogenic amine receptors from insects was composed of 59 sequences and 236 positions, being best described by a Blosum62+I+G model. The dataset encompassing vertebrate and in sect 5-HT receptors, including 5-HT2, contained 144 sequences and 263 positions, while the dataset with 5-HT2 receptor sequences excluded contained 113 sequences and 264 positions. These both were best described by a JTT+G model.

Phylogenic analyses were conducted using three different methods. Bayesian analysis was conducted using Mr. Bayes 3.1.2 to obtain the optimal tree topology and posterior probability values for the nodes. Analyses ran for 10^6^ MCMC generations and the burn-in value was estimated graphically by removing all trees prior to the plateau. Convergence was confirmed for all MCMC runs, with the final splits frequency remaining below 0.1. Maximum-likelihood trees and bootstrap values were obtained on 100 pseudoreplicate datasets using PhyML v.2.4.4 and RAxML 7.0.3. In all cases, nodes supported by greater than 0.8 posterior probability and 50% bootstrap support are considered noteworthy, while nodes with greater than 0.95 posterior probability and 80% bootstrap support are considered to be robust.

### Cell culture assays

The open reading frame of each of the receptors was subcloned into the pcDNA3.1 vector (Invitrogen) and transfected into HEK 293-EBNA cells (Invitrogen) using Fugene (Roche). Stable transformant lines were obtained by selecting for resistance to G418 according to manufacturer’s instructions (Invitrogen, Carlsbad CA). Cells were maintained in DMEM containing 10% FBS and 2mM glutamine. cAMP levels were measured using the cAMP dynamic2 cAMP HTRF measurement kit from Cisbio (Bedford Massachusetts) according to manufacturer’s instructions using a BioTek (Winooski, Vermont) Synergy 2 microplate reader. IP1 levels were measured using the IPOne HTRF assay from Cisbio according to manufacturer’s instructions [27]. Non-transfected control cells showed no responses to serotonin.

### Expression in *Xenopus* *oocytes*

The *Xenopus laevis* oocyte experiments were done as described previously [28]. Briefly, capped sense cRNA was prepared using the mMESSAGEmMACHINE T3 Kit (Ambion, UK) from linearized plasmid DNA containing full-length cDNAs encoding either the *Manduca sexta* 5-HT_1A_, 5-HT_1B_, 5-HT2 or 5-HT7 receptors in the pBS-MXT vector [29] generously provided by Dr. H.A. Lester. Sense cRNA was prepared in a similar manner from a Galpha16 (G16) clone in pCIH1 using the mMESSAGEmMACHINE T7 Kit (Ambion, UK) and from GIRK1 and GIRK2 clones in pBS-MXT using the mMESSAGEmMACHINE T3 Kit (Ambion, UK). In addition, sense cRNA was also prepared from a cDNA clone, pCF1, encoding the human cystic fibrosis channel, in pSP64 Poly(A) vector (Promega) using the mMESSAGEmMACHINE SP6 Kit (Ambion, UK).

Stage V and VI *X. laevis* oocytes were prepared as described previously [30]. They were injected with 50 ng of a receptor sense cRNA, either alone or with 0.5 ng each of GIRK1 and GIRK2 sense cRNA, or with 50 ng of Galpha16 sense cRNA, or with 50 ng of Cystic Fibrosis channel sense cRNA. Injected oocytes were incubated at 19 °C for 2-5 days before recording; uninjected oocytes were processed in parallel as controls.

Recordings were made using a two-microelectrode voltage-clamp technique, at a -60 mV holding potential, to measure oocyte currents [31]. Oocytes were continuously superfused with ND96 medium [30] and test substances were added to the superfusate. To assay the activation of GIRK channels, oocytes were voltage-clamped at -80 mV, equilibrated in high K^+^ medium (in mM: KCl 96 and NaCl 2 instead of KCl 2 and NaCl 96) to reverse the K^+^ ion gradient, and inward currents measured before, during, and after addition of the test substance. Drug treatment was terminated by washout with high K^+^ medium and subsequent switching to ND96 medium. To assay the cyclic AMP dependent activation of the cystic fibrosis channel, oocytes were voltage-clamped at -60mV and exposed to 10^-7^ M forskolin for 30 min before addition of test substances. Drug treatment was terminated by washout with forskolin containing medium and subsequent switching to ND96 medium. The dose response curves for each receptor were normalized based on the maximal average current evoked by the increasing concentrations of 5-HT and are plotted as non-linear regressions.

### Pharmacology

The following pharmacological agents were used in this study and were purchased from Sigma unless otherwise indicated; 5-hydroxtryptamine, 5-nonyloxytryptamine (5-N; Tocris), LP-44 (Tocris), epinephrine, norepinephrine, dopamine, octopamine, tyramine, histamine, 5-methoxytryptamine, 2-methyl-5-hydroxytryptamine, 8-hydroxy-DPAT, 5-carboxamidotryptamine, 8-hydroxy-PIPAT, BP554, CP94253, AS19, 2,5-dimethoxy-4-iodoamphetamine (DOI; otherwise known as 4-Iodo-2,5-dimethoxy-α-methylbenzene ethanamine), mianserin, prazosin, WAY100635, methiothepin, spioperone, and methysergide.

### Western Blots

Western blots were performed as described previously [32]. Briefly adult *Manduca* brains (1 brain per 4 lanes) were homogenized in NuPAGE LDS sample buffer (Invitrogen/Novex, Carlsbad, CA) containing dithiothreitol (Invitrogen) and protease-inhibitor cocktail (Sigma). Proteins were then separated on a NuPage 4–12% Bis-Tris polyacrylamide gradient gel in MOPS (3-[N-morpholino] propane sulfonic acid) buffer (Invitrogen) using the Novex electrophoresis system. The proteins were then transferred to PVDF membrane (Immobilon-P, Millipore) by using NuPAGE Transfer buffer (Invitrogen) and a Trans-Blot SD semidry transfer cell (Bio-Rad, Hercules, CA). The lanes were then separated using a scalpel and incubated in Tris-buffered saline (TBS) with 0.1% Tween (TBST) and 5% non-fat dry milk for 1 hour. The AM3A antibody (generously provided by Dr. M. Sosa) which was raised in rabbit against the 5-HT_1A_ receptor (5HT1MAC) of the fresh water prawn, *Macrobrachium* *rosenbergii* [33] was then added at a 1:3000 dilution and the blots were then incubated overnight at 4^o^C. The following day blots were washed in TBST, blocked in TBST with 5% milk and incubated with 1:5000 HRP-conjugated goat anti-rabbit antibody (Jackson ImmunoResearch; # 111-035-144) overnight at 4^o^C. The blots were then washed in TBS and developed with the Opti-4CN kit (4-chloro-1-naphthol substrate; BioRad).

### Immunocytochemistry

The brains of 2-5 day old moths were dissected (29 total spread across the different treatments described below) from the head capsules under physiological saline and placed in 4% paraformaldehyde overnight at 4^o^C. Brains were then washed in PBS (pH 6.9) embedded in 5% low melting point agarose (Sigma) and sectioned at 75µM thickness on a vibrating microtome (Technical Products International, St. Louis, MO). Sections were then washed in phosphate buffered saline (PBS) with 0.5% Triton-X 100 (PBST), blocked in 2% IgG-free bovine serum albumin (BSA) for one hour and then incubated overnight at 4^o^C in PBST with 2% BSA and a 1:300 dilution of the AM3A antibody. Sections were then washed in PBST, blocked as the day before and then incubated overnight at 4^o^C in 1:500 goat anti-rabbit FITC (Sigma). Sections were washed in PBST, PBS then 60% glycerol in PBS, and finally mounted on glass slides in 80% glycerol in PBS. For dual labeling with the AM3A antibody, a 1:500 dilution of mouse anti-GABA antibody (Abcam, Cambridge, MA) or a 1:500 dilution of goat anti-5-HT antibody (Immunostar, Hudson, WI) were included with the AM3A antibody with goat anti-mouse and donkey anti-goat (respectively) Cy5 secondary antibodies (both from Jackson Immunoresearch, Westgrove, PA) used the following day. On a technical note, we found that the AM3A antibody will only label tissue for a few days once thawed and stored at 4^o^C and the mouse anti-GABA antibody will not work if frozen regardless of cryoprotection. The full specificity controls for the lot of mouse anti-GABA antibody were described previously [34]. Staining with the goat anti-5-HT antibody was completely eliminated with pre-adsorption of the goat anti-5-HT antibody with 100 µg of serotonin/BSA conjugate (Immunostar product information sheet).

The immunogenic sequence from *Macrobrachium* *rosenbergii* used to generate the AM3A antibody was KDPDFLVRVNEHKKCLVSQD which is 65% identical to a portion of the Ms5HT1A receptor amino acid sequence; KDPDYLARITQQQKCLVSQD. The homologous sequence from *Manduca* was therefore used for pre-adsorption controls when the AM3A antibody was used for labeling *Manduca* tissue. For pre-adsorption controls, a 1:300 dilution of the AM3A antibody was incubated in PBST and 5µM of the *Manduca* homologous sequence (Genscript) overnight at 4^o^C. In parallel a 1:300 dilution of the AM3A antibody was incubated in PBST only overnight at 4^o^C. Both the pre-adsorbed and control solutions were then spun down for 15 minutes and the supernatants used to label *Manduca* brain tissue.

For the retrograde labeling of projection neurons, 2-5 day old moths were restrained in a plastic tube and their head-capsule opened exposing the brain. A minuten pin was dipped in Texas red, 3000 MW (Molecular Probes, Invitrogen, Carlsbad, CA) dissolved in PBS and then the dye covered pin was inserted into the brain near the dorso-posterior portion of the brain in the approximate location of the calyces of the mushroom bodies. The head capsule was then resealed and the incision in the cuticle coated in petroleum jelly to prevent desiccation. The moths were then placed at 4^o^C overnight to allow the dye to be transported and the following day the brain were dissected and treated as described above for labeling with the AM3A antibody.

Images were collected with a Zeiss 510 Meta laser scanning confocal microscope equipped with an argon laser and green and red HeNe lasers and appropriate filters. The Zeiss LSM Image Browser was used to create image stacks and to adjust contrast and brightness. CorelDRAW X4 (Corel Corporation) was used to organize all images and figures, and graphs were generated using GraphPad Prism v 5.01 (GraphPad Software, Inc.).

## Results

### Cloning of two new *Manduca sexta* 5-HT receptors and the phylogenetic relationships of all 4 Ms5HTRs

To date there are four known physiologically confirmed 5-HT receptor types expressed in insects; the 5-HT_1A_-like, 5-HT_1B_-like, 5-HT2-like and 5-HT7-like receptors [35]. The 5-HT_1A_ and B-like receptors from *Manduca* (Ms5HT1A and Ms5HT1B) were cloned previously [22]. Here we describe the cloning of the Ms5HT2 and Ms5HT7 receptors. Both gene products (1.9kb and 1.7kb lengths respectively) encode predicted 7-transmembrane domain proteins with predicted molecular weights of 70.4 and 63.2 kDa (respectively). Both sequences share high degrees of sequence identity with 5-HT2 and 7 receptors from other insect species (Figure 1). Figure 1 also highlights the 7 putative transmembrane domains and potential PKA, PKC, tyrosine kinase and N-glycosylation sites for both receptors.

**Figure 2:**
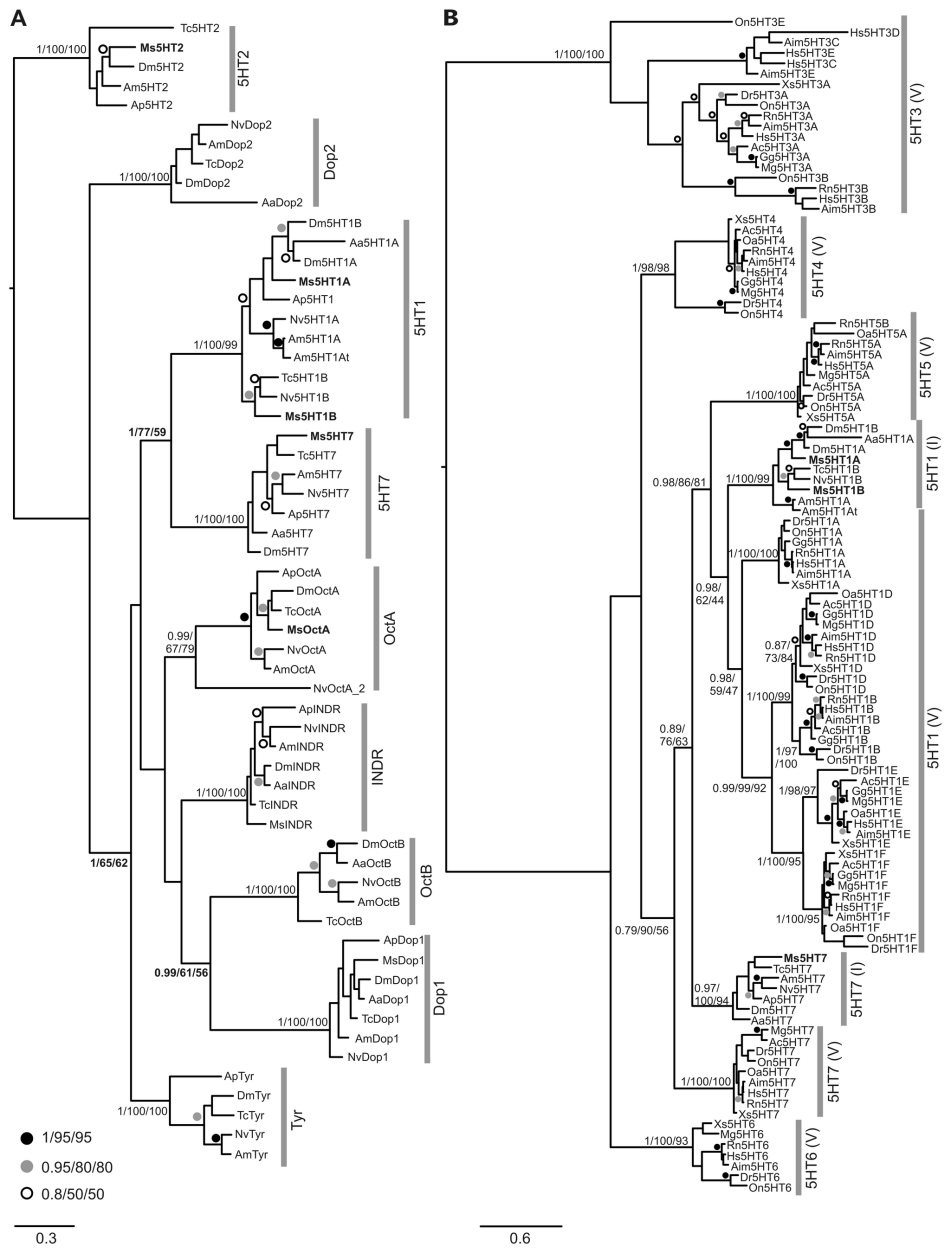
Phylogenetic relationships between the Ms5HTRs and other amine receptors. Ms5HTRs are indicated with bold text (A) Phylogenetic tree of all available serotonin, tyramine, octopamine and dopamine receptors from 7 species of insects. This figure is arbitrarily rooted on the 5-HT2 clade. Strongly supported clades for 5-HT1, 2, 7 Dopamine 1, 2, Octopamine A, B, and Tyramine are generated as shown. The newly characterized Ms5HT2R sequence is present in the 5-HT2 clade and Ms5HT7R sequence in the 5-HT7 clade. In this and all subsequent phylogenetic trees, the best Bayesian topology is shown with node support values listed in the order of Posterior probability values, Maximum Likelihood bootstrap support values for PhyML and Maximum Likelihood bootstrap support values for RAxML. Other values are replaced with symbols as shown in the figure. Vertical bars with labels denote the reconstructed clades. The scale bar represents the number of changes per site. (B) Phylogenetic analysis of serotonin receptors from insects (I) and vertebrates (V), with the 5-HT2 receptors excluded. The figure is arbitrarily rooted on the vertebrate 5-HT3 clade, but should be treated as unrooted. Note the reconstruction of the various named insect and vertebrate receptor sub-types.

We used phylogenetic analyses to address several questions regarding the evolutionary relationships of the *Manduca* 5-HT receptors. In our first analysis the genomes of seven insect species were searched to obtain potential homologs of the 5-HT1, 5-HT2 and 5-HT7 as well as all available tyramine, octopamine and dopamine receptors sequences. These sequences, together with the Ms5HTRs were combined into a single dataset and subjected to phylogenetic analysis. Our analysis produced 9 distinct, strongly supported clades for each of these receptor types by all 3 phylogenetic analysis methods used (Figure 2A). Importantly for their further characterization, the newly characterized Ms5HT2 and Ms5HT7 receptors, as well as the previously characterized Ms5HT1A and Ms5HT1B [22], all fell robustly within their proposed 5-HT clades, thus confirming their classification. To our surprise, there were also moderately supported nodes uniting the 5-HT2 and Dopamine 2-like receptor clades, the 5-HT1 and 5-HT7 receptor clades, as well as the Dop1, OctB clades. This has potential implications for biogenic amine receptor evolution, at least within the insects, as discussed below. As we also wanted to examine the extent of orthology between in sect 5-HT receptors and their vertebrate counterparts, we performed a series of phylogenic analyses on 5-HT receptor sequences of 7 species of insects and 10 species vertebrates. The purpose of this analysis was to describe the relationships between the different 5-HT receptors, so the 5-HT3 receptors was used as an outgroup rather than a G-protein coupled receptor that was not a 5-HT receptor. Our initial analysis showed a strongly supported grouping of vertebrate and in sect 5-HT2 (Figure S1), but less well-supported relationships otherwise. Since the 5-HT2 receptors appear evolutionarily disparate from the other 5-HT receptors (Figure 2A), we excluded them from our analysis. This produced a well-resolved phylogeny, showing both vertebrate-specific 5-HT subclasses and overall relationships between the insect and vertebrate 5-HT receptors (Figure 2B).

### Pharmacological Characterization of the four Ms5HT receptors

**Figure 3:**
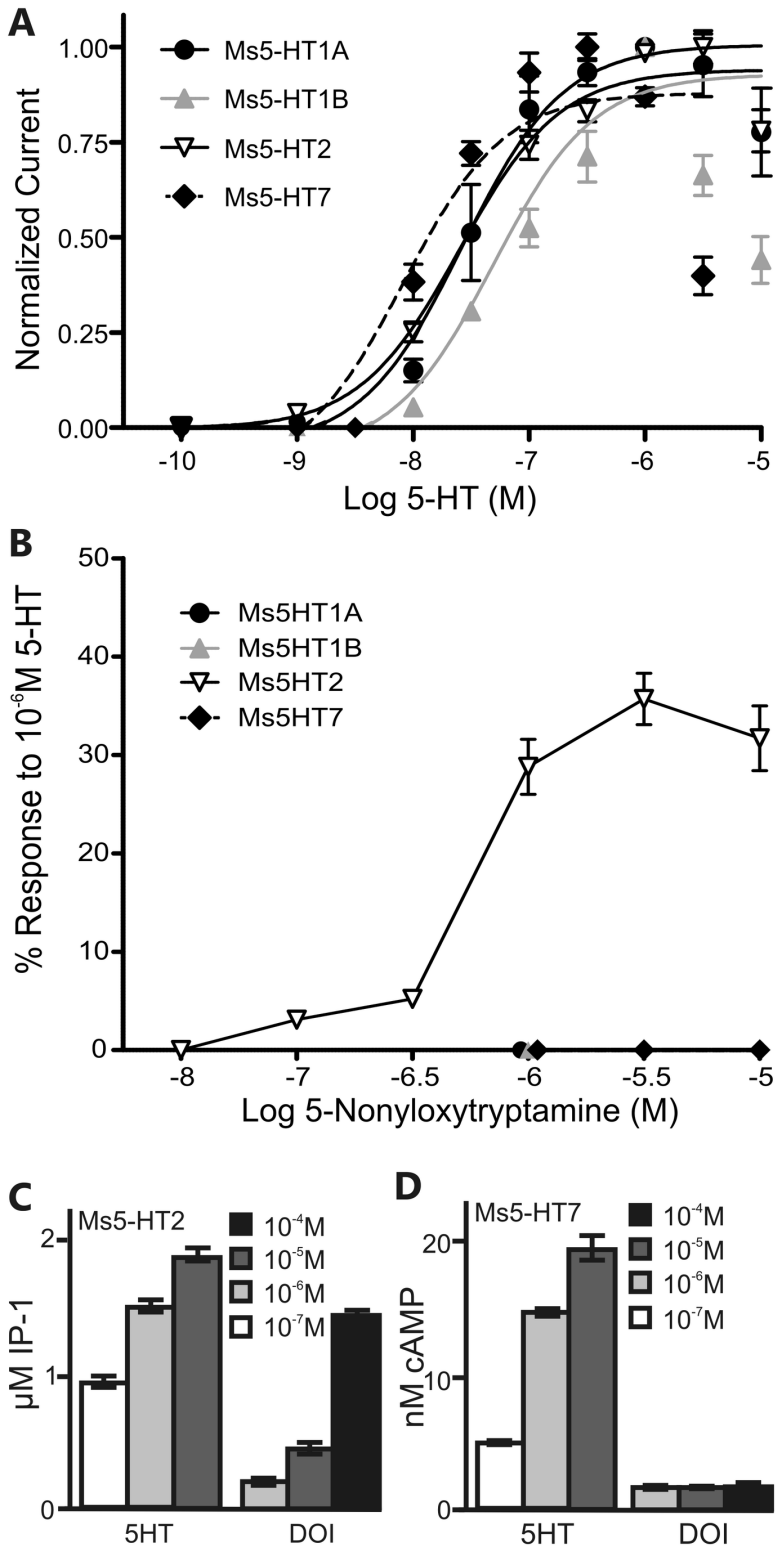
Responses of the Ms5HTRs for 5-HT and the dose response curves for selective agonists. (A) Currents (normalized to maximal responses) evoked by increasing concentrations of 5-HT (from 0.1nM to 10 µM) in *Xenopus laevis* oocytes expressing the Ms5HT1A (black circle), Ms5HT1B (grey triangle), Ms5HT2 (open triangle), Ms5HT7 (black diamond). (B) *Xenopus* oocytes expressing the Ms5HT2 receptor (open triangle) respond to 5-nonyloxytryptamine at 0.1 µM and above, while oocytes expressing the Ms5HT1A (black circle), Ms5HT1B (grey triangle) or Ms5HT7 (black diamond) receptors do not respond at any of the concentrations tested. The symbols for the Ms5HT1A, 1B and 7 receptors have been staggered for clarity. In (A) and (B) the 5HT_1A_ responses were measured in oocytes co-injected with receptor cRNA and cRNA for the CFTR channel whilst the 5HT_1B_, 5HT2 and 5HT7 responses were measured in oocytes injected with the receptor cRNA alone. Oocytes were exposed to 2 min pulses of the agonists at various concentrations. (C) HEK293 cells expressing the Ms5HT2 receptor respond (measured as nM concentration of IP-1/well of a 96 well plate) to 5-HT and DOI (4-Iodo-2,5-dimethoxy-α-methylbenzene ethanamine). (D) HEK293 cells expressing the Ms5HT7 receptor respond (measured as nM concentration of cAMP/well of a 96 well plate) to 5-HT, but not DOI. Shades of grey in (C) and D) indicate concentration of pharmacological agent applied. All error bars represent SEM.

**Figure 4:**
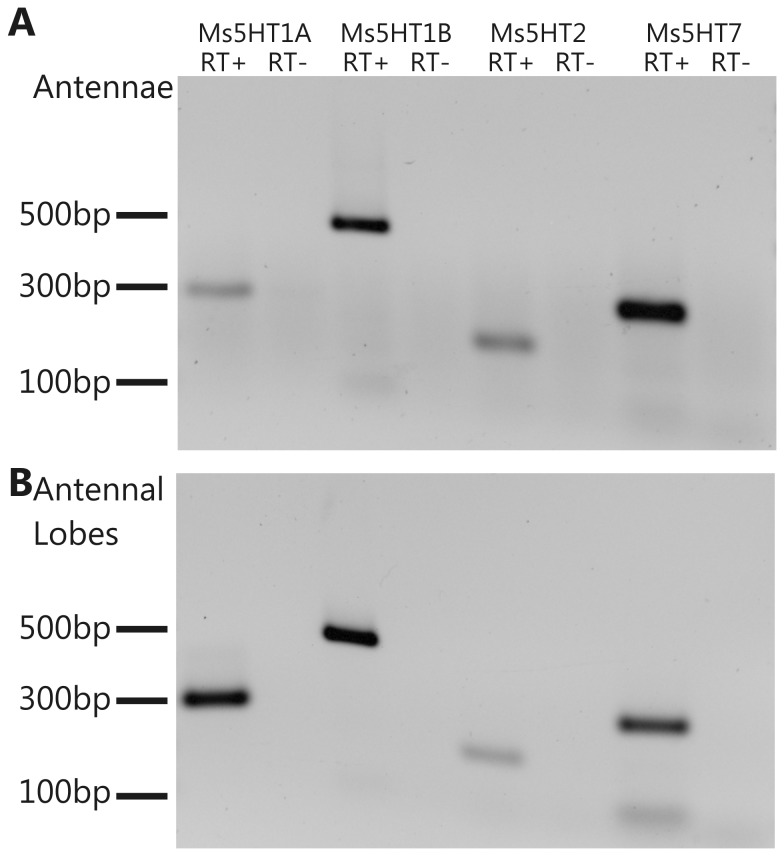
All four Ms5HTRs are expressed in both the antennal lobes and the antennae. RT-PCR for the four Ms5HTRs using cDNA from (A) antennal lobes and (B) the antennae. For each receptor, the left-hand lane depicts the RT-PCR with the reverse transcriptase included during cDNA production and right-hand lane depicts the RT-control to ensure no genomic DNA contamination.

**Figure 5:**
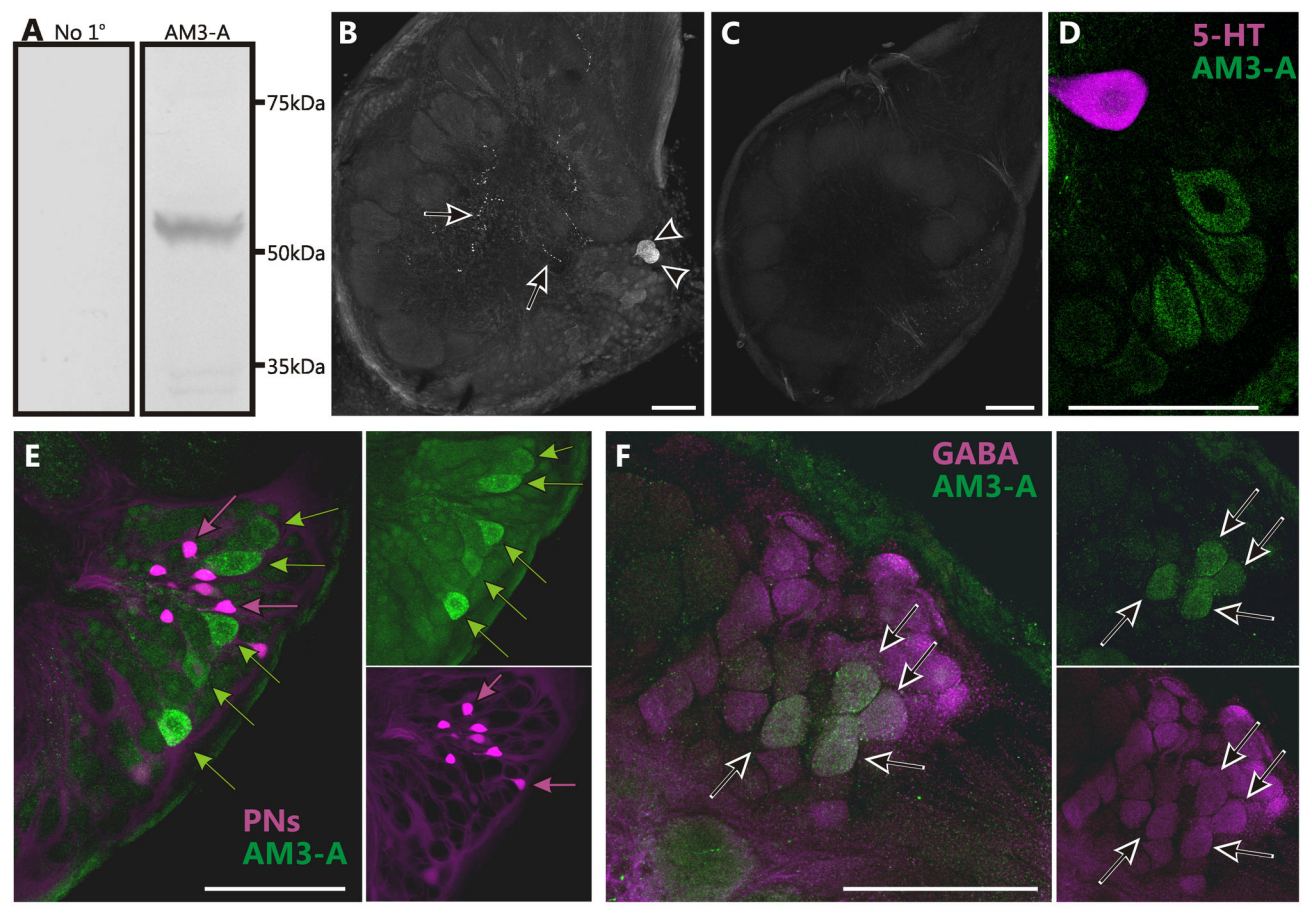
The Ms5HT1A receptor is expressed in a subset of GABA-like immunoreactive local interneurons. (A) Western blots of the AM3-A antibody against *Manduca* protein and in which the AM3-A antibody was omitted (no 1^o^) produced no labeling. (B) Frontal section through an AL labeled using an antibody against a 5-HT_1A_ receptor from prawn (AM3-A) generously provided by Dr. Maria Sosa. Note cell bodies (arrowheads; 24±2) and fine processes (arrows). (C) *Manduca* ALs labeled with AM3-A antibody pre-adsorbed with the Ms5HT1A receptor sequence (KDPDYLARITQQQKCLVSQD) homologous to the antigenic sequence used to generate the AM3-A antibody (KDPDFLVRVNEHKKCLVSQD) resulted in no labeling. (D) Double labeling against the Ms5HT1A receptor (green) and 5-HT (magenta) show no overlap. (E) Backfills of projection neurons (PNs) (magenta) reveal no co-localization of the Ms5HT1A receptor (green) in the lateral cell cluster of the AL. Magenta arrows indicate PNs and green arrows indicate Ms5HT1A-ir neurons. (F) Double labeling against the Ms5HT1A receptor (green) and GABA (magenta) revealed a subset of Ms5HT1A-ir cell bodies co-localizing GABA (arrows). All scale bars=100µm.

To determine if the putative 5-HT receptors do in fact respond selectively to 5-HT and not to other amines, we expressed all four receptors in *Xenopus* oocytes and tested their responses to 5-HT, norepinephrine, epinephrine, dopamine, octopamine, tyramine and histamine (Table 1) at a concentration of 1µM. The Ms5HT1A responses were measured in oocytes co-injected with receptor cRNA and cRNA for the CFTR channel suggesting that this receptor couples to changes in cAMP levels when expressed in oocytes. In contrast, the responses to the Ms5HT1B, Ms5HT2 and Ms5HT7 receptors were measured in oocytes injected with the receptor cRNA alone suggesting that they can couple to the activation of the endogenous, inward calcium-dependent chloride current of the oocyte. With the exception of the Ms5HT1A receptor, none of the Ms5HTRs responded to any amine other than 5-HT. The Ms5HT1A receptor gave relatively weak responses to norepinephrine and epinephrine at 17% and 3.3% (respectively) of the response to 5-HT. However, norepinephrine and epinephrine are not expressed at significant levels in the nervous system of *Manduca* [36], so the Ms5HT1A receptor can be considered selective for 5-HT with respects to endogenously produced amines. Previous studies have reported concentration dependent effects of 5-HT on AL neurons, which could be due to differences in the relative potency of 5-HT for the Ms5HTRs. We therefore established the dose response curves for all of the Ms5HTRs (Figure 3A) to 5-HT. The EC50 values were all within one log unit of each indicating that 5-HT had a similar potency for activating each receptor; Ms5HT1A EC_50_ = 29.5nM, Ms5HT1B EC_50_ = 57.0nM, Ms5HT2 EC_50_ = 25.4nM, Ms5HT7 EC_50_ = 10.5nM.

**Table 1:** Amine Specificity *Manduca* 5HT Receptors.

	**5HT_1A_ Receptor**	**n**	**5HT_1B_ Receptor**	**n**	**5HT 2 Receptor**	**n**	**5HT 7 Receptor**	**n**
**5HT**	100% (143.9 ± 14.7 nA)	6	100% (124.9 ± 10.0 nA)	12	100% (319.7 ± 35.5 nA)	8	100% (297.8 ± 31.5 nA)	15
**Epinephrine**	17.1±9.6%	3	0	4	0	3	0	4
**Norepinephrine**	3.3 ±1.6%	3	0	4	0	3	0	3
**Dopamine**	0	4	0	3	0	3	0	3
**Octopamine**	0	5	0	3	0	3	0	3
**Tyramine**	0	3	0	3	0	3	0	3
**Histamine**	0	3	0	3	0	3	0	4

The 5HT_1A_ responses were measured in oocytes coinjected with receptor cRNA and cRNA for the CFTR channel whilst the 5HT_1B_, 5HT2 and 5HT7 responses were measured in oocytes injected with the receptor cRNA alone. Oocytes were exposed to 2 min pulses of the agonists at a concentration of 1 µM. Results are expressed as a percentage ± SEM of the response of the same oocytes to 2 min control pulses of 5HT.

We next examined the effects of various pharmacological agents on the individual Ms5HTRs using the *Xenopus* oocyte expression system for the Ms5HT1A and B receptors and the HEK-293 cell expression system for the Ms5HT2 and 7 receptors. Table 2 summarizes the effects of a variety of 5-HT receptor agonists tested at 1µM on the Ms5HT1A and 1B receptors. Surprisingly few agonists activated the Ms5HT1A or B receptors with the exception of 5-methoxytryptamine which weakly activated both receptors. Upon testing an array of 5-HT receptor antagonists (summarized in Table 3) on the Ms5HT1A and B receptors we found that WAY-100635 and methiothepin antagonized the Ms5HT1B receptor, while WAY-100635 only weakly antagonized the Ms5HT1A receptor. The Ms5HT2 receptor was activated at concentrations of 5-nonyloxytryptamine as low as 0.1 µM, whereas the other 3 receptors were not activated at 1 µM and in the case of the Ms5HT7 receptor, 10 µM (Table 2 and Figure 3B). The pharmacological agent 2,5-dimethoxy-4-iodoamphetamine (DOI) is well established as being highly selective for vertebrate 5-HT2 receptors so we sought to determine if DOI also activated the Ms5HT2R. However, because of restrictions on the use of DOI in the United Kingdom, experiments testing the effects of DOI were performed at the University of Arizona using the HEK-293 expression system (Figure 3C, D). When expressed in HEK 293 cells, the Ms5HT2 receptor mediated changes in the release of intracellular calcium levels which were assessed as changes in IP-1 production and the Ms5HT7 receptor mediated changes in intracellular cAMP levels. DOI activated Ms5HT2 receptors expressed in HEK293 cells at a concentration of 10µM, which is consistent with previous findings that DOI activates the Drosophila 5-HT2 receptor [37], yet did not activate the Ms5HT7 receptor even at a concentration of 100µM. Finally, we tested the effects of methysergide, which has been found to antagonize all four 5-HT receptors in *Drosophila* [37,38], on the Ms5HTRs expressed in *Xenopus* oocytes. *Xenopus* oocytes expressing the Ms5HT1A, 2 and 7 receptors were stimulated with 3 µM methysergide, 1 µM 5-HT or a combination of 3µM methysergide and 1 µM 5-HT. Surprisingly, methysergide alone agonized the Ms5HT1A and 7 receptors (Table 4). Furthermore, there was very little difference in the level of activation between the 5-HT alone and 5-HT in combination with methysergide treatments. This is consistent with recent findings on the effects of methysergide on the 5-HT2 and 5-HT7 receptors of the blowfly [39].

**Table 2:** The effect of synthetic Agonists on the Ms5HT1A and Ms5HT1B receptors.

**Drug**	**Receptor Subtype Specificity**	**5HT_1A_ receptor**	**n**	**5HT_1B_ receptor**	**n**
**5HT**	Non-specific	100% (158.3 ± 17.4 nA)	23	100% (100.5 ± 17.6 nA)	30
**5-Methoxytryptamine**	Non-specific	20.8 ± 6.7%	5	7.2 ± 1.4%	7
**2-Methyl-5-hydroxy-tryptamine**	Non-specific	3.9 ± 2.2%	3	0	4
**8-Hydroxy DPAT**	Non-specific	0	4	0	3
**5-Carboxamido-tryptamine**	Non-specific	0	3	0	3
**8-Hydroxy-PIPAT**	5HT_1A_	0	3	0	4
**BP554**	5HT_1A_	0	3	0	4
**5-Nonyloxy-tryptamine**	5HT_1B_	0	3	0	7
**CP94253**	5HT_1B_	0	3	0	4
**AS19**	5HT7	0	3	0	3
**LP44**	5HT7	0	3	0	3

The 5HT_1A_ responses were measured in oocytes coinjected with receptor cRNA and cRNA for the CFTR channel while the 5HT_1B_ responses were measured in oocytes injected with the receptor cRNA alone. Oocytes were exposed to 2 min pulses of the agonists at a concentration of 1 µM. Results are expressed as a percentage ± SEM of the response of the same oocytes to 2 min control pulses of 5HT.

**Table 3:** The effect of synthetic Antagonists on the Ms5HT1A and Ms5HT1B receptors.

**Drug**	**Receptor Subtype Specificity**	**5HT_1A_ receptor**	**n**	**5HT_1B_ receptor**	**n**
**5HT**	Non-specific	100% (150.5 ± 14.2 nA)	14	100% (100.5 ± 17.6 nA)	30
**WAY100635**	5HT_1A_	76.8 ± 15.8%	3	38.2 ± 3.3%	7
**Methiothepin**	5HT1,5HT6,5HT7	111.7 ± 2.7%	3	36.4 ± 12.6%	7
**Spiperone**	5HT2A	116.3 ± 15.7%	5	82.4 ± 16.5%	4
**Mianserin**	Non-specific	112.3 ± 3.5%	3	103.0 ± 3.6%	3
**Prazosin**	Non-specific	124.1 ± 14.2%	8	93.0 ± 18.9%	8

The 5HT_1A_ responses were measured in oocytes coinjected with receptor cRNA and cRNA for the CFTR channel while the 5HT_1B_ responses were measured in oocytes injected with the receptor cRNA alone. Oocytes were exposed to a 2 min pulse of 1 µM 5-HT in the presence of antagonists at a concentration of 3 µM. Oocytes were preincubated for 5 min in antagonist before 5-HT application. The results are expressed as a percentage of the response to a control pulse of 1 µM 5-HT in the same oocytes in the absence of antagonists.

**Table 4:** Effects of Methysergide on the Ms5HT1A, 2 and 7 receptors.

**Drug**	**Ms5HT1A**	**n**	**Ms5HT2**	**n**	**Ms5HT7**	**n**
**Methysergide alone**	27.8 ± 8%	6	0%	6	49.5 ± 12%	6
**5-HT alone**	100% (137.0 ± 6.5nA)	6	100% (382.3 ± 37.9nA)	6	100% (328.0 ± 14.7 nA)	6
**Methysergide + 5-HT**	101.0 ± 27%	6	110.1 ± 24%	6	87.8 ± 16%	6

The 5HT_1A_ responses were measured in oocytes coinjected with receptor cRNA and cRNA for the CFTR channel while the 5HT2 and 5HT7 responses were measured in oocytes injected with the receptor cRNA alone. Oocytes were exposed to 2 minute pulses of either 3µM methysergide, 1µM 5-HT or 3µM methysergide and 1µM 5-HT. Results are expressed as a percentage ± SEM of the response of the same oocytes to 2 min control pulses of 5HT.

### The Ms5HT1A receptor is expressed in a population of GABAergic antennal lobe local interneurons

Although all of the glomeruli of the AL of *Manduca* are innervated by a single serotonergic neuron [40], the effects of 5-HT on AL neuron responses are heterogeneous. Previous studies have reported that not all AL neurons are affected by 5-HT [5,8,41] and that the effects of 5-HT in the AL can be odor dependent [5,12]. There are two possible, non-exclusive explanations for the diverse effects of 5-HT. The first is that AL neurons are themselves highly diverse by nature and therefore the effects of 5-HT accentuate the physiological differences already present between groups of AL neurons. The second is that the diversity of effects of 5-HT is due to the heterogeneous expression of the different Ms5HT receptors by the diverse population of AL neurons. Therefore, knowing the patterns of expression for the Ms5HT receptors is crucial for explaining the diversity of effects of 5-HT.

We first sought to determine if all of the Ms5HT receptors are expressed within the olfactory system. We therefore performed RT-PCR for all of the receptors using cDNA generated from the antennae (Figure 4A) and the ALs (Figure 4B). The RT-PCR detected cDNA for all four receptors from both tissue preparations indicating that mRNA for all of the receptors is likely to be expressed within the first two levels of the olfactory system. We next sought to determine the expression pattern of the Ms5HT1A receptor using immunocytochemistry. We were able to successfully label the nervous tissue of *Manduca* using an antibody raised against the 5-HT_1A_ receptor of the freshwater prawn *Macrobrachium* *rosenbergii* [33]. In Western blots against *Manduca* nervous tissue, this antibody produced a single band at 51kDA (Figure 5A) which is the estimated molecular weight of the Ms5HT1A receptor [22]. Immunocytochemical staining of *Manduca* AL sections resulted in labeling of approximately 24 ± 2 cell bodies (n=6) in the lateral cell packet and sparse punctate labeling throughout the AL neuropil (Figure 5B). This labeling was eliminated (n=4) by pre-adsorbing the antibody with the peptide sequence from *Manduca* that was homologous to the antigenic sequence from *Macrobrachium* *rosenbergii* (Figure 5C). The single serotonergic CSD neuron [40,42] which provides 5-HT-ir innervation to the AL does not express the Ms5HT1A receptor (Figure 5D; n=4) indicating that in the AL the Ms5HT1A receptor does not function as an autoreceptor. Retrograde fills of projection neuron (the output neurons of the AL) axons from the mushroom bodies revealed no instances of overlap between the projection neurons and the Ms5HT1A receptor (Figure 5E; n=7) and the Ms5HT1AR-ir cell bodies were relatively large compared to projection neuron cell bodies. Finally, double labeling against the Ms5HT1A receptor and GABA revealed that almost all Ms5HT1AR-ir cell bodies were also GABA-ir (Figure 5F; n=8). These data suggest that the Ms5HT1A receptor is expressed primarily by a subset of GABAergic local interneurons.

## Discussion

The consequences of neuromodulation in neural networks are complicated by the diversity of neuromodulatory receptors expressed. Functional populations of neurons express different suites of receptors that may be coupled to a range of different second messenger pathways and because these groups of neurons play distinct roles in information processing, the consequences for the release of a neuromodulator can be extensive and multifaceted. In insects, 5-HT is involved in a vast array of different behaviors from sensory processing to motor output, demonstrating the ubiquity of its influences on nervous system function. Furthermore, individual in sect 5-HT receptors have been demonstrated to play functional roles in the regulation of circadian rhythms [43–45], aggression [46], learning [47,48], courtship [49], insulin signaling [50], salivary secretion [39] and phototaxis [51,52]. Both the diversity of behaviors affected and the ubiquity of serotonergic innervation in the brains of insects highlight the critical role 5-HT plays in normal brain function. In this study we began to examine the receptor basis for serotonergic modulation of olfactory processing in the antennal lobe (AL) of the moth *Manduca sexta*. To this end we cloned all four in sect 5-HT receptor subtypes from *Manduca*, examined their phylogenetic relationships with other insect amine receptors and vertebrate 5-HT receptors, pharmacologically characterized the *Manduca* 5-HT receptors and determined the expression pattern of one of the receptors in the AL of *Manduca*.

Our phylogenetic analyses grouped all four Ms5HTRs with strong support to the four 5-HT receptor type clades from other insects (Figure 2). This analysis suggested that the in sect 5-HT1 and 7 type receptors are more closely related compared to the 5-HT2 receptors, consistent with previous phylogenetic analyses [35,53,54]. Our analysis of biogenic amine receptors in insects showed resolution in the tree topology uniting receptors of different specificity. This suggests that, whatever the original substrate-specificity of the receptor, it has not remained stable over evolutionary time, i.e. there was not a single 5-HT receptor that gave rise to 5-HT1, 2, and 7 to the exclusion of dopamine, octopamine and tyramine receptors. Finally, we performed two analyses to confirm and extend past results relating to the orthology of the vertebrate and insect receptors. We do find that the 5HT2 receptors are clearly orthologous and while the 5-HT7 and 5-HT1 receptors of insects and vertebrates are broadly orthologous, there are also several vertebrate-specific subclasses that are interspersed within them, consistent with previous findings [53,55,56]. However, there are named 5-HT1 A and B subclasses in both insects and vertebrates. These do not appear to be orthologous, but instead the results of two separate and independent duplication events (Figure 2B). Thus, these should not be treated as equivalent in the literature (i.e. a vertebrate 5-HT_1A_ receptor is not orthologous to the in sect 5-HT1A receptor).

The pharmacological assays demonstrated that the Ms5HTRs were all selective for 5-HT over other amines (Table 1) confirming that, based on a physiological criteria, they are 5-HT receptors. Although the coupling of receptors to second messengers in expression systems does not necessarily reflect the G-protein coupling of the receptors in vivo, we can make some observations as to the second messenger systems potentially activated by the Ms5HT receptors. In the HEK293 expression system, the Ms5HT2 receptor elevated IP3 levels and the Ms5HT7 receptor elevated cAMP levels suggesting that they couple to Gq and Gs respectively in this system, consistent with biochemical studies of the 5-HT2 [37] and 5-HT7 [38,57] receptors in other insect species. However, in *Xenopus* oocytes both the Ms5HT2 and Ms5HT7 receptors couple to the activation of endogenous inwardly rectifying chloride channels via a calcium dependent pathway. This suggests that either the Ms5HT7 receptor can couple to different second messenger pathways in a cell specific manner or perhaps that in the HEK293 cells its ability to elevate cAMP levels might depend on the presence of a calcium sensitive adenylyl cyclase. The pharmacological assays also revealed that while methysergide acts as a pan-serotonin receptor antagonist in *Drosophila* [37,38], it agonized the Ms5HT1A and 7 receptors with little to no antagonistic effects on the Ms5HTRs (Table 4). These partial agonist effects of methysergide are highly consistent with those reported for the 5-HT2 and 7 receptors from the blowfly [58].

In the AL, 5-HT has concentration dependent effects, with low concentrations decreasing the strength of AL neuron responses, and higher concentrations enhancing AL neuron responses [8]. However, all four Ms5HTRs had similar EC50 values ranging from 10.5nM to 57.0nM, so differences in receptor activation thresholds is not a sufficient explanation for this concentration dependency. The receptors could be expressed at different levels within a single cell or different levels of activation of a single receptor could activate different second messenger systems with opposing effects on neuronal excitability. Different levels of Ca^2+^ influx via NMDA receptors result in either LTD or LTP [59] and agonist specific activation of different second messenger systems has been observed for the tyramine/octopamine receptor of Drosophila [60] demonstrating that the manner in which a single receptor is activated can differentially activate second messenger systems. Finally, concentration dependent effects of 5-HT could arise from the expression of the individual receptors by different functional neuronal populations. The concentration of 5-HT required to induce direct modulation of a given neuron relative to the concentration of 5-HT required to produce a noticeable change in the influence of the synaptic input from other AL neurons could differ markedly. Lateral excitation and inhibition in the ALs of *Drosophila* differ in their odor concentration thresholds for activation [61], so it is conceivable that populations of neurons that differ in their functional roles could also differ in the threshold at which 5-HT modulation of their activity becomes apparent in the activity of other cells.

The expression pattern for a suite of receptors dictates the consequences of neuromodulation within a circuit. Based on our immunocytochemical data, we hypothesize that the Ms5HTRs are likely heterogeneously expressed within the AL by different functional subtypes of neurons, similar to the distribution of 5-HT receptors in distinct sub-divisions of the central complex of *Drosophila* [45,50,62]. Because the Ms5HT1A receptor was expressed in a small subset of GABA-ir local interneurons and yet previous studies in *Manduca* have found that ~50% of AL neurons are affected by 5-HT [5,6,8], a homogeneous pattern of expression in which every neuron affected by 5-HT expresses the same suite of Ms5HT receptors appears unlikely. It is possible that the Ms5HTRs are expressed in a very small number of neurons that modulate AL activity, making the effects of 5-HT on projection neurons indirectly mediated. Although the effects of 5-HT on local interneurons have been confirmed to be direct in cell culture [10,41], projection neurons have not been similarly studied and so the effects of 5-HT on projection neurons could be due to the modulation of odor-evoked lateral interactions between neurons. Because local interneurons are far from a monolithic population [63–66], the consequences of serotonergic modulation of these neurons for odor processing are likely to be complex. Future studies will be directed at identifying the expression patterns of the other three Ms5HT receptors once additional antibodies can be identified.

The release of neuromodulators within a specific physiological context is a widely observed phenomenon. For instance, the serotonergic neurons from the dorsal and median Raphe nuclei project throughout the brain [17,67,68] and vary in their activity depending upon the waking-state of the animal [17–21], whereas the dopaminergic neurons from the ventral tegmental area are active within the context of reward (reviewed in 69). The consequences of the release of these modulators is made extremely complex due to the number of receptor types expressed and the diversity in the physiological properties and functional roles of the neuronal types within a given neural network. For instance, pyramidal neurons in Layer V of the prefrontal cortex express both 5-HT_1A_ and 5-HT2A receptors [70–72]. While activation of the 5-HT_1A_ receptor induces a hyperpolarization of pyramidal cells [70,71], 5-HT2A receptor cause a slow depolarization [70–73]. These seemingly contradictory effects suggest that viewing the activation of a given 5-HT receptor as either excitatory or inhibitory is an oversimplification. The consequences of the activation of each receptor differ in timescale allowing 5-HT to modulate the gain of the pyramidal cells (via the 5-HT2A receptor) while also limiting the range (via the 5-HT_1A_ receptor) within which the cells respond to synaptic input, thus making the cells respond more strongly and selectively [74]. Furthermore, activation of the 5-HT2A receptor in this system also causes glutamate spillover adding an additional layer of complexity to the consequences of neuromodulation within this network [75–77]. By examining a neuromodulatory system in a numerically less complex neural network, we hope to shed light on organizational principles conserved across taxa and regions of the brain.

## Supporting Information

Figure S1 Phylogenetic analysis of serotonin receptors from insects (**I**) and vertebrates (**V**). The figure is arbitrarily rooted on the vertebrate 5-HT3 clade, but should be treated as unrooted. Note the reconstruction of the 5-HT2 clade uniting both insect and vertebrate versions of these receptors.
(TIF)

Table S1 **List of all receptor sequences used for phylogenetic analysis.**
(DOCX)
