# Supplementary material for: A Characterization of the Manduca sexta Serotonin Receptors in the Context of Olfactory Neuromodulation
Source: PLoS One. 2013 Jul 29;8(7):e69422. doi: 10.1371/journal.pone.0069422 (PMC3726668; doi:10.1371/journal.pone.0069422)
Supplement: Table S1 — List of all receptor sequences used for phylogenetic analysis. (DOCX) [file pone.0069422.s002.docx]

**Table S1: List of all receptor sequences used for phylogenetic analysis**

| **Species** | **Receptor type** | **Name in Fig. 2** | **Published name** | **Accession number** |
| --- | --- | --- | --- | --- |
| **Insects** |  |  |  |  |
| *Acyrthosiphon pisum* | 5HT1 | Ap5HT1 | 5HTp | XP_001949725 |
|  | 5HT2 | Ap5HT2 | 5HT2 | XP_001947553 |
|  | 5HT7 | Ap5HT7 | 5HT1 | XP_003241835 |
|  | OctA | ApOctA | Oct1 | XP_001948930 |
|  | INDR | ApINDR | Dop2 | XP_003241369 |
|  | Dop1 | ApDop1 | Dop1 | XP_001947683 |
|  | TyrR | ApTyr | Octp | XP_001944003 |
| *Aedes aegypti* | 5HT1 | Aa5HT1A | 5HT1A | XP_001653194 |
|  | 5HT7 | Aa5HT7 | 5HT7 | XP_001661980 |
|  | INDR | AaINDR | OAMB | XP_001651499 |
|  | Dop2 | AaDop2 | Dopp | XP_001647989 |
|  | OctB | AaOctB | OctB | XP_001651713 |
| *Apis mellifera* | 5HT1 | Am5HT1A | 5HT1A | NP_001164579 |
|  | 5HT1 | Am5HT1At | 5HT1t | NP_001164579.1 |
|  | 5HT2 | Am5HT2 | 5HT2 | CBX90120 |
|  | 5HT7 | Am5HT7 | 5HT7 | NP_001071289 |
|  | Dop1 | AmDop1 | Dop1 | NP_001011595 |
|  | INDR | AmINDR | INDR | NP_001011567 |
|  | Dop2 | AmDop2 | Dop2 | NP_001014983 |
|  | OctA | AmOctA | Oct | NP_732541 |
|  | OctB | AmOctB | OctB | XP_396348 |
|  | TyrR | AmTyr | TyrR | NP_001011594 |
| *Drosophila melanogaster* | 5HT1 | Dm5HT1A | 5HT1A | CAA77570 |
|  | 5HT1 | Dm5HT1B | 5HT1B | NM_079065 |
|  | 5HT2 | Dm5HT2 | 5HT2 | NP_730859 |
|  | 5HT7 | Dm5HT7 | 5HT7 | NP_524599 |
|  | Dop1 | DmDop1 | Dop1 | U22106 |
|  | INDR | DmINDR | DAMB | U61264 |
|  | Dop2 | DmDop2 | Dop2 | NM_001014760 |
|  | OctA | DmOctA | OctA | NP_732541 |
|  | OctB | DmOctB | OctB | NP_001034049 |
|  | TyrR | DmTyr | TyrR | NP_524419 |
| *Manduca sexta* | 5HT1 | Ms5HT1A | 5HT1A | DQ840515 |
|  | 5HT1 | Ms5HT1B | 5HT1B | DQ840516 |
|  | 5HT2 | Ms5HT2 | 5HT2 | JX891652 |
|  | 5HT7 | Ms5HT7 | 5HT7 | JX878498 |
|  | Dop1 | MsDop1 | Dop1 | JN117928 |
|  | INDR | MsINDR | INDR | JN117929 |
|  | OctA | MsOctA | OctA | DQ840514 |
| *Nasonia vitripennis* | 5HT1 | Nv5HT1A | 5HT2 | XP_003425665 |
|  | 5HT1 | Nv5HT1B | 5HTp | XP_001603891 |
|  | 5HT7 | Nv5HT7 | 5HT1 | XP_001606275.2 |
|  | Dop1 | NvDop1 | Dop1 | XP_001606438 |
|  | INDR | NvINDR | Dop2 | NP_001155849 |
|  | Dop2 | NvDop2 | Dop2p | XP_001602510 |
|  | OctA | NvOctA | OctA | XP_003427475 |
|  | OctA | NvOctA_2 | 5HT7 | XP_003426713 |
|  | OctB | NvOctB | OctB | XP_001606684 |
|  | TyrR | NvTyr | TyrRp | XM_003426524 |
| *Tribolium castaneum* | 5HT1 | Tc5HT1B | 5HT1B | XP_972856 |
|  | 5HT2 | Tc5HT12 | 5HT2 | XP_972327 |
|  | 5HT7 | Tc5HT7 | 5HT7 | XP_966577 |
|  | Dop1 | TcDop1 | Dop1 | XM_966449 |
|  | INDR | TcINDR | INDR | XM_967686 |
|  | Dop2 | TcDop2 | Dop2 | XP_969037 |
|  | OctA | TcOctA | Octp | XP_970007 |
|  | OctB | TcOctB | OctB | XP_974238 |
|  | TyrR | TcTyr | TyrR | NP_001164311 |
| **Vertebrates** |  |  |  |  |
| *Anolis carolinensis* | 5HT1B | Ac5HT1B |  | XP_003215747 |
|  | 5HT1D | Ac5HT1D |  | XP_003230616 |
|  | 5HT1E | Ac5HT1E |  | XP_003215587 |
|  | 5HT1F | Ac5HT1F |  | XP_003219185 |
|  | 5HT2A | Ac5HT2A |  | XP_003215394 |
|  | 5HT2C | Ac5HT2C |  | XP_003226755 |
|  | 5HT3A | Ac5HT3A |  | XP_003226221 |
|  | 5HT4 | Ac5HT4 |  | XP_003217396 |
|  | 5HT5A | Ac5HT5A |  | XP_003221972 |
|  | 5HT7 | Ac5HT7 |  | XP_003225512 |
| *Ailuropoda melanoleuca* | 5HT1A | Aim5HT1A |  | XP_002926351 |
|  | 5HT1B | Aim5HT1B |  | XP_002926886 |
|  | 5HT1D | Aim5HT1D |  | XP_002919578 |
|  | 5HT1E | Aim5HT1E |  | XP_002924747 |
|  | 5HT1F | Aim5HT1F |  | XP_002926879 |
|  | 5HT2A | Aim5HT2A |  | XP_002915023 |
|  | 5HT2B | Aim5HT2B |  | XP_002917486 |
|  | 5HT2C | Aim5HT2C |  | XP_002924537 |
|  | 5HT3A | Aim5HT3A |  | XP_002926667 |
|  | 5HT3B | Aim5HT3B |  | XP_002928458 |
|  | 5HT3C | Aim5HT3C |  | XP_002915711 |
|  | 5HT3E | Aim5HT3E |  | XP_002915737 |
|  | 5HT4 | Aim5HT4 |  | XP_002930602 |
|  | 5HT5A | Aim5HT5A |  | XP_002926094 |
|  | 5HT6 | Aim5HT6 |  | XP_002915696 |
|  | 5HT7 | Aim5HT7 |  | XP_002917335 |
| *Danio rerio* | 5HT1A | Dr5HT1A |  | NP_001116793 |
|  | 5HT1B | Dr5HT1B |  | NP_001122181 |
|  | 5HT1D | Dr5HT1D |  | NP_001139158 |
|  | 5HT1E | Dr5HT1E |  | XP_002665689 |
|  | 5HT1F | Dr5HT1F |  | XP_001344430 |
|  | 5HT2A | Dr5HT2A |  | XP_688270 |
|  | 5HT2B | Dr5HT2B |  | NP_001038208 |
|  | 5HT2C | Dr5HT2C |  | XP_001339040 |
|  | 5HT3A | Dr5HT3A |  | XP_700338 |
|  | 5HT3C | Dr5HT3C |  | XP_003201188 |
|  | 5HT4 | Dr5HT4 |  | XP_001337671 |
|  | 5HT5A | Dr5HT5A |  | NP_001007122 |
|  | 5HT6 | Dr5HT6 |  | XP_696681 |
|  | 5HT7 | Dr5HT7 |  | XP_690599 |
| *Gallus gallus* | 5HT1A | Gg5HT1A |  | NP_001163999 |
|  | 5HT1B | Gg5HT1B |  | NP_001166252 |
|  | 5HT1D | Gg5HT1D |  | XP_001232312 |
|  | 5HT1E | Gg5HT1E |  | XP_001235179 |
|  | 5HT1F | Gg5HT1F |  | XP_425535 |
|  | 5HT2A | Gg5HT2A |  | XP_425628 |
|  | 5HT2C | Gg5HT2C |  | XP_426265 |
|  | 5HT3A | Gg5HT3A |  | XP_425806 |
|  | 5HT4 | Gg5HT4 |  | XP_414481 |
| *Homo sapiens* | 5HT1A | Hs5HT1A |  | NM_000524 |
|  | 5HT1B | Hs5HT1B |  | NM_000863 |
|  | 5HT1D | Hs5HT1D |  | NM_000864 |
|  | 5HT1E | Hs5HT1E |  | NM_000865 |
|  | 5HT1F | Hs5HT1F |  | NM_000866 |
|  | 5HT2A | Hs5HT2A |  | NM_000621 |
|  | 5HT2B | Hs5HT2B |  | NM_000867 |
|  | 5HT2C | Hs5HT2C |  | NM_000868 |
|  | 5HT3A | Hs5HT3A |  | NM_213621 |
|  | 5HT3B | Hs5HT3B |  | NM_006028 |
|  | 5HT3C | Hs5HT3C |  | NM_130770 |
|  | 5HT3D | Hs5HT3D |  | NM_001145143 |
|  | 5HT3E | Hs5HT3E |  | NM_182589 |
|  | 5HT4 | Hs5HT4 |  | NM_000870 |
|  | 5HT5 | Hs5HT5 |  | NM_024012 |
|  | 5HT6 | Hs5HT6 |  | NM_000871 |
|  | 5HT7 | Hs5HT7 |  | NM_000872 |
| *Meleagris gallopavo* | 5HT1D | Mg5HT1D |  | XP_003212537 |
|  | 5HT1E | Mg5HT1E |  | XP_003204409 |
|  | 5HT1F | Mg5HT1F |  | XP_003202888 |
|  | 5HT2A | Mg5HT2A |  | XP_003203398 |
|  | 5HT2A2 | Mg5HT2A2 |  | XP_003203395 |
|  | 5HT2C | Mg5HT2C |  | XP_003208363 |
|  | 5HT3A | Mg5HT3A |  | XP_003212768 |
|  | 5HT4 | Mg5HT4 |  | XP_003210342 |
|  | 5HT5A | Mg5HT5A |  | XP_003207002 |
|  | 5HT6 | Mg5HT6 |  | XP_003212353 |
|  | 5HT7 | Mg5HT7 |  | XP_003206102 |
| *Ornithorhynchus anatinus* | 5HT1D | Oa5HT1D |  | XP_001514834 |
|  | 5HT1E | Oa5HT1E |  | XP_001513201 |
|  | 5HT1F | Oa5HT1F |  | XP_001515164 |
|  | 5HT2A | Oa5HT2A |  | XP_001514168 |
|  | 5HT2B | Oa5HT2B |  | XP_001508933 |
|  | 5HT4 | Oa5HT4 |  | XP_001507325 |
|  | 5HT5A | Oa5HT5A |  | XP_001512760 |
|  | 5HT7 | Oa5HT7 |  | XP_001505726 |
| *Oreochromis niloticus* | 5HT1A | On5HT1A |  | XP_003446125 |
|  | 5HT1B | On5HT1B |  | XP_003453310 |
|  | 5HT1D | On5HT1D |  | XP_003453267 |
|  | 5HT1F | On5HT1F |  | XP_003445408 |
|  | 5HT2A | On5HT2A |  | XP_003449594 |
|  | 5HT2C | On5HT2C |  | XP_003453988 |
|  | 5HT3A | On5HT3A |  | XP_003453775 |
|  | 5HT3B | On5HT3B |  | XP_003453774 |
|  | 5HT3E | On5HT3E |  | XP_003459689 |
|  | 5HT4 | On5HT4 |  | XP_003454279 |
|  | 5HT5A | On5HT5A |  | XP_003439268 |
|  | 5HT6 | On5HT6 |  | XP_003449184 |
|  | 5HT7 | On5HT7 |  | XP_003455723 |
| *Rattus Norvegicus* | 5HT1A | Rn5HT1A |  | NP_036717 |
|  | 5HT1B | Rn5HT1B |  | NP_071561 |
|  | 5HT1D | Rn5HT1D |  | NP_036984 |
|  | 5HT1F | Rn5HT1F |  | NP_068629 |
|  | 5HT2A | Rn5HT2A |  | NP_058950 |
|  | 5HT2B | Rn5HT2B |  | NP_058946 |
|  | 5HT2C | Rn5HT2C |  | NP_036897 |
|  | 5HT3A | Rn5HT3A |  | NP_077370 |
|  | 5HT3B | Rn5HT3B |  | NP_071525 |
|  | 5HT4 | Rn5HT4 |  | NP_036985 |
|  | 5HT5A | Rn5HT5A |  | NP_037280 |
|  | 5HT5B | Rn5HT5B |  | NP_077371 |
|  | 5HT6 | Rn5HT6 |  | NP_077341 |
|  | 5HT7 | Rn5HT7 |  | NP_075227 |
| *Xenopus silurana* | 5HT1A | Xs5HT1A |  | XP_002934244 |
|  | 5HT1D | Xs5HT1D |  | XP_002938881 |
|  | 5HT1E | Xs5HT1E |  | XP_002933964 |
|  | 5HT1F | Xs5HT1F |  | XP_002931817 |
|  | 5HT2A | Xs5HT2A |  | XP_002935159 |
|  | 5HT2C | Xs5HT2C |  | XP_002936408 |
|  | 5HT3A | Xs5HT3A |  | XP_002937868 |
|  | 5HT4 | Xs5HT4 |  | XP_002939852 |
|  | 5HT5A | Xs5HT5A |  | XP_002932543 |
|  | 5HT6 | Xs5HT6 |  | XP_002942005 |
|  | 5HT7 | Xs5HT7 |  | XP_002940056 |

The species, receptor type, abbreviations and accession numbers for all of the receptor sequences used in the phylogenetic analyses described in Figure 2 and S1.
